# The Homburg‐Adelaide toric IOL nomogram: How to predict corneal power vectors from preoperative IOLMaster 700 keratometry and total corneal power in toric IOL implantation

**DOI:** 10.1111/aos.16742

**Published:** 2024-07-16

**Authors:** Achim Langenbucher, Nóra Szentmáry, Jascha Wendelstein, Alan Cayless, Peter Hoffmann, Michael Goggin

**Affiliations:** ^1^ Department of Experimental Ophthalmology Saarland University Homburg/Saar Germany; ^2^ Dr. Rolf M. Schwiete Center for Limbal Stem Cell and Aniridia Research Saarland University Homburg/Saar Germany; ^3^ Department of Ophthalmology Semmelweis‐University Budapest Hungary; ^4^ Department of Ophthalmology Johannes Kepler University Linz Linz Austria; ^5^ School of Physical Sciences The Open University Milton Keynes UK; ^6^ Augen‐ und Laserklinik Castrop‐Rauxel Castrop‐Rauxel Germany; ^7^ Faculty of Health and Medical Sciences University of Adelaide Adelaide South Australia Australia; ^8^ The Queen Elizabeth Hospital Adelaide South Australia Australia

**Keywords:** feedforward neural network, keratometric power, multilinear regression, statistical correction models, toric intraocular lenses, total corneal power, vector analysis

## Abstract

**Purpose:**

The purpose of this study is to compare the reconstructed corneal power (RCP) by working backwards from the post‐implantation spectacle refraction and toric intraocular lens power and to develop the models for mapping preoperative keratometry and total corneal power to RCP.

**Methods:**

Retrospective single‐centre study involving 442 eyes treated with a monofocal and trifocal toric IOL (Zeiss TORBI and LISA). Keratometry and total corneal power were measured preoperatively and postoperatively using IOLMaster 700. Feedforward neural network and multilinear regression models were derived to map keratometry and total corneal power vector components (equivalent power EQ and astigmatism components C0 and C45) to the respective RCP components.

**Results:**

Mean preoperative/postoperative C0 for keratometry and total corneal power was −0.14/−0.08 dioptres and −0.30/−0.24 dioptres. All mean C45 components ranged between −0.11 and −0.20 dioptres. With crossvalidation, the neural network and regression models showed comparable results on the test data with a mean squared prediction error of 0.20/0.18 and 0.22/0.22 dioptres^2^ and on the training data the neural network models outperformed the regression models with 0.11/0.12 and 0.22/0.22 dioptres^2^ for predicting RCP from preoperative keratometry/total corneal power.

**Conclusions:**

Based on our dataset, both the feedforward neural network and multilinear regression models showed good precision in predicting the power vector components of RCP from preoperative keratometry or total corneal power. With a similar performance in crossvalidation and a simple implementation in consumer software, we recommend implementation of regression models in clinical practice.

## 1 BACKGROUND

Corneal power or refraction could be described either in a standard notation in terms of the meridional power (in dioptres) and axis (in degrees) in the flat and steep meridian or using power vector components. These components include the equivalent power (EQ) and the astigmatism (C), defined as the difference between power values in the steep and flat meridian projected to the 0 and 90 degree (C0) and to the 45‐ and 135‐degree axis (C45). Since in regular astigmatism the flat and steep meridian are assumed to be orthogonal, providing either the steep or flat axis is sufficient. Both the standard and the power vector component notation are equivalent and can be interconverted algebraically.

Calculation of toric intraocular lenses can be based on several different combinations of measures. These include: the raw keratometric data characterising the curvature of the anterior corneal curvature only; the keratometric data with a statistical correction for the corneal back surface astigmatism (Chen et al., 2022; LaHood et al., 2017, 2018; Langenbucher, Hoffmann, et al., 2024); the corneal power data from the anterior and posterior surface considering the cornea as thick meniscus lens; or the total corneal power data derived from a Scheimpflug tomographer or optical coherence tomography (Koch et al., 2013; Savini et al., 2017; Tutchenko et al., 2020). In this context, the systematic portion of the astigmatic change in the cornea induced by cataract surgery (e.g. from corneal incisions) could be considered by taking the surgically induced astigmatism (SIA) into account. However, the SIA resulting from modern paralimbal small incisions in the cornea is quite small and may be neglected in most cases, even if there is some stochastic variation in the topographic change of the cornea with cataract surgery (Reitblat et al., 2015; Tutchenko et al., 2020).

For over 100 years, it has been well known in ophthalmology that the keratometric power is not fully representative of the refraction properties of the cornea (Asiedu et al., 2016; Elliott et al., 1994; Grosvenor et al., 1988; Koch et al., 2012). The average ratio of anterior to posterior corneal curvature is quite similar under physiological conditions without any history of corneal surgery (Langenbucher, Szentmáry, Cayless, Weisensee, et al., 2022). However, this can change after corneal surgery with the consequence that the equivalent power of the cornea may not be properly represented by keratometry. Also, in general, the anterior to posterior corneal curvature ratio is not the same for all meridians (Langenbucher et al., 2023; Langenbucher, Taroni, et al., 2024). Typically, the posterior corneal surface adds some extra astigmatism against the rule (ATR, steep axis of anteriorly measured astigmatism with an orientation between 0 and 30° or between 150 and 180°) and this is not described with keratometry restricted to a corneal front surface measurement only (Asiedu et al., 2016; Elliott et al., 1994; Grosvenor et al., 1988; Koch et al., 2012, 2013). The consequence is that front surface keratometry tends to overestimate with‐the‐rule corneal astigmatism (WTR, steep axis of astigmatism with an orientation between 60 and 120°) and to underestimate ATR astigmatism.

This is mostly relevant in calculation of toric intraocular lenses as the cylindric power would need to be corrected to account for this mismatch. In addition to measuring the total corneal power (e.g. total keratometry of the IOLMaster 700), we could also back‐calculate total corneal power (reconstructed corneal power, RCP) in terms of equivalent power and astigmatism from a reliable postoperative refraction at the spectacle plane, the spherocylindrical lens power and measured orientation in the eye. This RCP will differ from total corneal power values derived from device measurement, as it also it is influenced by the SIA and potential induction of refractive cylinder resulting from misalignment of the lens orientation in terms of decentration and tilt (Langenbucher et al., 2023; Langenbucher, Hoffmann, et al., 2024; Langenbucher, Szentmáry, Cayless, Weisensee, et al., 2022; Langenbucher, Taroni, et al., 2024). It will also be influenced by non‐corneal and non‐IOL sources of refractive astigmatism which can influence the patients' choices during manifest refraction. The positioning of the toric lens is mostly self‐controlled by the capsular bag configuration.

Using keratometry without statistical correction instead of total corneal power or RCP values for toric lens calculation results on average in overestimation of lens cylinder values with WTR or underestimation of lens cylinder in ATR astigmatism. Therefore, we expect a residual ATR astigmatism in the postoperative refraction. Where the astigmatism is purely WTR or ATR, keratometry could be corrected for toric lens power calculation. However, with oblique astigmatism (OBL, steep axis of astigmatism with an orientation between 30 and 60° or between 120 and 150°), some authors do not recommend a correction of keratometry for posterior corneal astigmatism (Sheen Ophir et al., 2020). If surgeons prefer total corneal power data for toric lens calculation, data from modern optical tomographers could be directly used. However, if RCP is preferred, then a representative dataset with previous cataract surgeries and toric lens implantation with postoperative refraction data and lens power and orientation must be analysed to calculate a prediction model which maps the preoperative keratometry or total corneal power to RCP (Alpins & Goggin, 2004).

The purposes of the present study were

- to compare power vector components EQ, C0 and C45 of keratometry and total corneal power from a modern optical biometer with the back‐calculated RCP power vector components at the corneal plane derived from postoperative refraction and power/orientation of the toric intraocular lenses in a large dataset with cataract surgeries with toric lens implantation,
- to derive feedforward neural shallow network based and regression‐based prediction models which map keratometry and total corneal power vector components to the respective RCP power vector components, and
- to analyse the performance of these prediction models in a cross‐validation setting.

## 2 METHODS

### 2.1 Dataset for our study and surgical details

A dataset with *N* = 442 clinical data entries from The Queen Elizabeth Hospital and Ashford Advanced Eye Care (Adelaide, Australia) was considered for this retrospective study. All data were anonymised at source and stored in a. XLSX file, which was transferred to the Department of Experimental Ophthalmology for further analysis. Data tables were reduced to the relevant parameters required for our analysis, consisting of patient age in years, gender (male or female), laterality of the eye (OS or OD), and the corresponding measurement parameters as reported by each device/measurement modality in the study as listed below, together with the specifications of the implanted toric lenses in each case:

IOLMaster 700 (Carl‐Zeiss‐Meditec, Jena, Germany): Axial length (AL) in mm, anterior chamber depth (ACD) in mm (considered from the corneal epithelium to the front apex of the crystalline lens), preoperative/postoperative keratometric power in the flat meridian (KF in dioptres at KA in degrees) and in the steep meridian (KS in dioptres), and preoperative/postoperative total keratometry as a measure for total corneal power in the flat meridian (TKF in dioptres at TKA in degrees) and in the steep meridian (TKS in dioptres).

Manual refraction: Sphere (REFS in dioptres) and cylinder (REFC in dioptres at REFA in degrees) measured with trial lenses in a trial frame at a refraction lane distance of 6 m. Step sizes of 0.25 dioptres were used for both the spherical and the cylindrical lenses. Visual acuity was recorded at lane distance both with (CDVA) and without (UDVA) correction.

### 2.2 Toric intraocular lens

Toric lens model with plate haptics (Carl Zeiss Meditec (Jena, Germany); either monofocal lens AT TORBI 709, *N* = 282; or trifocal lens AT LISA 939, *N* = 160), labelled spherical equivalent power (IOLSEQ in dioptres) and cylindrical lens power (IOLC in dioptres at target axis IOLTA in degrees and postoperatively measured axis IOLMA in degrees, indicating the (marked) flat axis of the toric lens).

Eyes with missing or incomplete data in any of the above‐mentioned values were excluded at source. All eyes were measured before cataract surgery with the IOLMaster 700, and at 4–8 weeks postoperatively with the IOLMaster 700, manual refraction and slit lamp measurement to evaluate IOLMA.

All surgeries were performed between June 2018 and November 2022 by an experienced surgeon (MG) under local anaesthesia. After para‐limbal 1.8 mm micro incision from the temporal side at the measured horizontal axis, the anterior chamber was filled with a cohesive OVD, with the creation of a continuous curvilinear capsulorhexis slightly smaller than the optical diameter of the lens (approximately 5.25 mm). After a standard phacoemulsification procedure, the toric lens was inserted and aligned with the anterior steep corneal meridian as measured by the IOLMaster 700 device. Special care was taken to remove all viscoelastic behind and surrounding the IOL and to ensure that the corneal incision and both paracenteses were hydrated. The Institutional Review Board provided a waiver for this study (Ärztekammer des Saarlandes, 157/21). Informed consent of the patients was not required. The study followed the tenets of the Declaration of Helsinki.

### 2.3 Pre‐processing of the data

The data were transferred to Matlab (Matlab 2022b, MathWorks, Natick, USA) for further processing. Custom software was written in Matlab to decompose the preoperative and postoperative keratometric data (IOLMaster 700 KF/KS/KA), total corneal power data (IOLMaster 700 TKF/TKS/TKA), toric lens power data (labelled IOLEQ/IOLC and target axis IOLTA or measured axis IOLMA), and refraction data (REFS/REFC/REFA) into power vector components EQ, C0 and C45 (Alpins & Goggin, 2004; Langenbucher et al., 2023; Langenbucher, Szentmáry, Cayless, Weisensee, et al., 2022). To account for lateral symmetry of the power vectors, the power vector components for the oblique axis C45 were flipped in sign for left eyes to consider all eyes as right eyes (Langenbucher, Szentmáry, Cayless, Röggla, et al., 2022; Langenbucher, Taroni, et al., 2024). The defocus equivalent DEQ was taken as an overall quality metric for refractive outcome after toric lens implantation and was derived from the power vector components of spectacle refraction by: $\mathrm{DEQ}=\sqrt{{\text{REFEQ}}^{2}+\frac{1}{4}∙\text{REFC}0^{2}+\frac{1}{4}∙\text{REFC}45^{2}}$. The defocus equivalent is commonly used when dealing with corneal astigmatism or refractive cylinder as it is known to correlate with the loss of visual acuity.

### 2.4 Reconstruction of the vergence deficit at the corneal plane RCP

For this calculation, we assume a simplified pseudophakic eye model having 3 refracting surfaces: a thin lens spectacle refraction at vertex distance of 12 mm in front of the cornea; a thin lens cornea; a thin lens tIOL at an effective lens position (ELP) behind the cornea, and with the focal plane at axial length behind the cornea. For the interspace between spectacle correction and cornea, we assumed a refractive index of *n* = 1.0. Between the cornea and the toric IOL, we assumed aqueous humour with *n*_A_ = 1.336, and between the toric IOL and the focal plane we assumed vitreous humour with *n*_V_ = 1.336 (Liou & Brennan, 1997). The ELP was derived according to the Haigis formula (Haigis et al., 2000) based on a linear regression with an intercept a_0_ and scaling a_1_ for the ACD and a_2_ for AL. For the formula constants, we used a_0_/a_1_/a_2_ = 0.912/0.4/0.1 for the TORBI 709 and 0.96/0.4/0.1 for the AT LISA respectively, as listed in IOLCon (https://IOLCon.org, accessed on May 10, 2024). Back‐calculation of RCP works in one of two ways: (A) using forward vergence transformation from the object space to the corneal plane, and (B) using backward vergence transformation from the focal plane to the corneal plane. (A): Assuming the object plane to be 6 m in front of the spectacle plane the object vergence at the spectacle plane is −1/6 dioptres. After adding the postoperative spectacle refraction, we obtain the vergence behind the spectacle plane. This vergence is transformed through the vertex distance and gives us the vergence in front of the corneal plane. (B) For backward vergence transformation, we assume a spherical vergence (AL‐ELP)/*n*_V_ behind the plane of the toric lens. After subtraction of the labelled spherocylindrical lens power, we obtain the vergence in front of the plane of the toric lens. This vergence is transformed backwards through the ELP to obtain the vergence behind the corneal plane. In the last step, RCP is calculated by subtracting the vergence in front of the corneal plane (result of A) from the vergence behind the corneal plane (result of B). Again, RCP is decomposed into power vector components RCPEQ, RCPC0 and RCPC45.

### 2.5 Prediction models to map power vector components to RCP

Two different setups were used as prediction models to map the power vector components of preoperative keratometry and total corneal power to the respective RCP vector components: (A) a feedforward shallow neural network structure NET with 2 hidden layers and 12/8 neurons for the first/second layer (Langenbucher et al., 2023). For the cost function, we used the mean squared prediction error (MSE); and (B) a multilinear regression based prediction REG implemented as a maximum likelihood estimator with an iterative ECM algorithm (Expectation/Conditional Maximisation algorithm) (Meng et al., 1993; Sexton & Swensen, 2000). The respective results are described in terms of LogL as the value of the log likelihood objective function after the final iteration and the root‐mean‐squared value of the Euclidean vector norm of the residuals as a measure for the prediction performance (Langenbucher, Szentmáry, Cayless, Weisensee, et al., 2022; Meng et al., 1993; Sexton & Swensen, 2000). NET_Keratometry_ and REG_Keratometry_ describe the predictions of RCP from preoperative keratometry with a neural network and linear regression, whereas NET_TotalCornealPower_ and REG_TotalCornealPower_ describe the corresponding predictions of RCP from preoperative total corneal power. The dataset was split randomly into a training set and a test set with a ratio of 70%–30%. The training set was used for setting up the NET and REG and calculating the weights and biases of NET and the independent test set was used to evaluate the performance of the predictions.

### 2.6 Statistical analysis and data presentation

Data are listed exploratively in terms of the arithmetic mean, standard deviation (SD), median, and the lower and upper boundaries of the 95% confidence interval (2.5% and 97.5% quantiles). The astigmatism components C0 and C45 of the power vectors were analysed using double angle plots showing the C0/C45 vector component in the *X*/*Y* axis. For correlation of UDVA with the DEQ, absolute value of REFEQ and REFC the Pearson correlation coefficient R was derived. A significance level of *p* < 0.05 was considered statistically significant. Error ellipses for the 95% confidence intervals were extracted from the variance–covariance matrices and the centroids and areas of the error ellipses (derived from the eigenvalues and eigenvectors) indicating the data scatter were documented.

## 3 RESULTS

The mean age of the *N* = 442 (57% female) patients was 71.05 ± 12.02 years (median 72 years). 51% of the eyes were right eyes. Table 1 lists the most relevant explorative data in terms of mean, standard deviation, median, and the lower and upper boundaries of the 95% confidence interval for biometric measures AL, ACD, for the power of the toric intraocular lens IOLEQ and IOLC, for the postoperative refraction REFEQ and REFC, and for the postoperative uncorrected and corrected distance visual acuity UDVA and CDVA.

**TABLE 1:** Explorative listing of the most relevant preoperative biometric measurements: Axial length AL, anterior chamber depth ACD (measured from the corneal epithelium to the front apex of the crystalline lens) as derived with the IOLMaster 700, (labelled) equivalent and toric power of the implanted toric lens (IOLEQ and IOLC), postoperative refraction (spherical equivalent REFEQ, cylinder REFC and defocus equivalent DEQ), and postoperative uncorrected (UDVA) and corrected distance visual acuity (CDVA). SD and 2.5%/97.5% quantile refer to the standard deviation and the lower and upper bounds of the 95% confidence interval respectively.

*N* = 442	AL in mm	ACD in mm	IOLEQ in D	IOLC in dpt	REFEQ in D	REFC in D	DEQ in D	UDVA in LogMAR	CDVA in LogMAR
Mean	23.6688	3.1219	20.1804	1.8179	−0.0716	0.3935	0.4774	0.1195	0.0284
SD	1.2167	0.4232	3.6260	0.9590	0.6331	0.3840	0.5036	0.1886	0.1412
Median	23.5200	3.1300	20.7500	1.5000	0.0000	0.2500	0.3536	0.0969	0.0000
2.5% quantile	21.7385	2.2900	9.7500	1.0000	−1.2500	0.0000	0.0000	−0.1984	−0.1984
97.5% quantile	26.9550	3.9045	26.6125	4.5000	0.9312	1.2500	1.5207	0.6021	0.3979

Table 2 shows the power vector components EQ, C0 and C45 for the preoperative and postoperative keratometry and total corneal power. It can be seen that preoperative and postoperative mean C0 vector component of the total corneal powers is systematically shifted by around 0.16 D towards ATR (more negative values) compared to keratometry.

**TABLE 2:** Power vector components of preoperative and postoperative keratometry and total corneal power derived from the IOLMaster 700 data. EQ refers to the equivalent power, C0 to the projections of the astigmatism to the 0°/90° meridian, and C45 to the projections of the astigmatism to the 45°/135° meridian. To account for lateral symmetry of the power vectors, the C45 power vector components were flipped in sign for left eyes to consider all eyes as right eyes. SD and 2.5%/97.5% quantile refer to the standard deviation and the lower and upper bounds of the 95% confidence interval respectively.

*N* = 442, data in dioptres	Preoperative keratometry			Preoperative total corneal power			Postoperative keratometry			Postoperative total corneal power		
	EQ	C0	C45	EQ	C0	C45	EQ	C0	C45	EQ	C0	C45
Mean	43.7843	−0.1401	−0.1152	43.8474	−0.3049	−0.1591	43.8466	−0.0772	−0.1582	43.9077	−0.2369	−0.2004
SD	1.5160	1.2984	0.6970	1.5107	1.3294	0.7083	1.5417	1.3361	0.7149	1.5398	1.3497	0.7438
Median	43.8125	−04547	−0.1614	43.8150	−0.5886	−0.1754	43.8250	−0.2367	−0.1596	43.8650	0.3746	−0.1779
2.5% quantile	41.0237	−2.4823	−1.3914	41.1706	−2.6123	−1.4805	41.0845	−2.5799	−1.6180	41.1050	−2.7645	−1.6528
97.5% quantile	47.0457	2.3333	1.1509	47.0937	2.1381	1.1041	47.0976	2.4841	1.2279	47.0720	2.4596	1.3082

**FIGURE 1:**
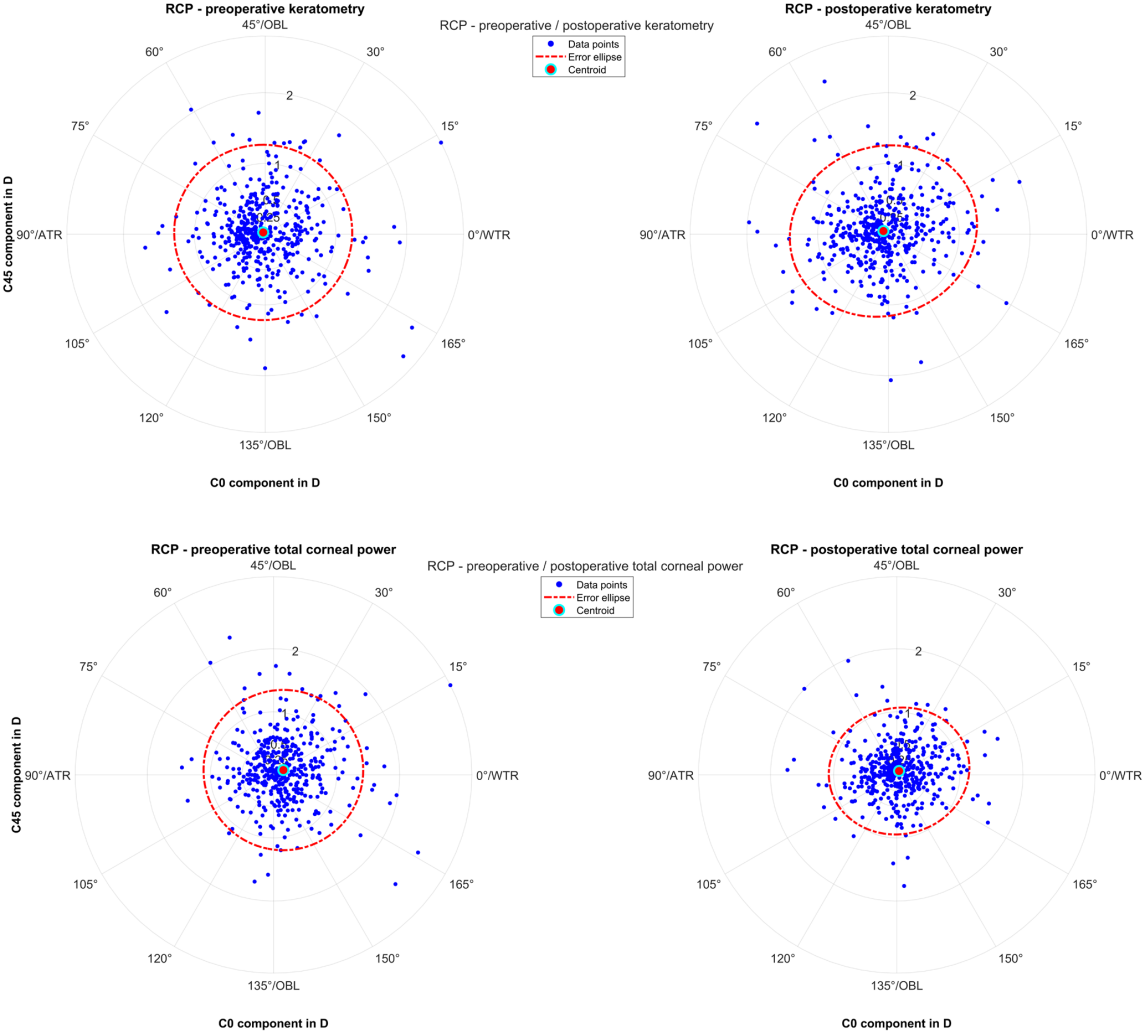
Double angle plot showing the astigmatism power vector components (C0 and C45: Projection to the 0/90° and to the 45°/135° meridian) for the differences between the reconstructed corneal power RCP and the preoperative/postoperative keratometry (upper row) and preoperative/postoperative total corneal power measured (lower row) as measured with the IOLMaster 700. The error ellipses (red dash‐dotted lines) together with the red filled circle markers refer to the 95% error ellipses and the centroids of the bivariate distribution. WTR/ATR/OBL respectively refers to the orientation of astigmatism with‐the‐rule/against‐the‐rule/oblique.

Table 3 lists the power vector components EQ, C0 and C45 for the differences between RCP and the preoperative and postoperative keratometry and total corneal power. It is clear from the data that the standard deviations of all 3 power vector components are smaller for the differences between RCP and postoperative keratometry and (especially) total corneal power as compared to the respective standard deviations for the differences between RCP and preoperative keratometry and total corneal power. This means that the RCP generally matches better to the postoperative measurements, especially to the total corneal power data. Figure 1 displays the respective differences in the astigmatism power vector components C0 and C45 (RCP minus preoperative and postoperative keratometry and total corneal power) in double angle plots. It can be seen directly from the graphs that the area of the 95% error ellipse is smallest for the difference RCP minus postoperative corneal power followed by RCP minus postoperative keratometry. This indicates that RCP matches more closely with the postoperative measured corneal power, especially with the total corneal power.

**TABLE 3:** Differences in the power vector components between reconstructed corneal power RCP and preoperative and postoperative keratometry and total corneal power derived from the IOLMaster 700 data. EQ refers to the equivalent power, C0 to the projections of the astigmatism to the 0°/90° meridian, and C45 to the projections of the astigmatism to the 45°/135° meridian. To account for lateral symmetry of the power vectors, the power vector components for the oblique axis C45 were flipped in sign for left eyes to consider all eyes as right eyes. SD and 2.5%/97.5% quantile refer to the standard deviation and the lower and upper bounds of the 95% confidence interval respectively.

*N* = 442, data in dioptres	RCP – Preoperative keratometry			RCP – Preoperative total corneal power			RCP – Postoperative keratometry			RCP – Postoperative total corneal power		
	EQ	C0	C45	EQ	C0	C45	EQ	C0	C45	EQ	C0	C45
Mean	0.0896	−0.1378	0.0254	0.0245	0.0562	0.0766	0.0286	−0.1181	0.0627	−0.0360	0.0371	0.0617
SD	0.4927	0.5138	0.5061	0.48853	0.5169	0.5193	0.3626	0.5412	0.4949	0.3156	0.2639	0.2384
Median	0.0966	−0.2078	0.0034	0.0374	0.0150	0.0547	−0.0176	−0.1436	0.0306	−0.0593	0.0118	0.0526
2.5% quantile	−0.8596	−1.0760	−1.0556	−0.9434	−0.8276	−0.9360	−0.6741	−1.1013	−0.9649	−0.6314	−0.4787	−0.4524
97.5% quantile	0.9938	1.0245	1.2885	0.9090	1.3530	1.2589	0.7637	1.1140	1.2380	0.6286	0.5844	0.5504

**FIGURE 2:**
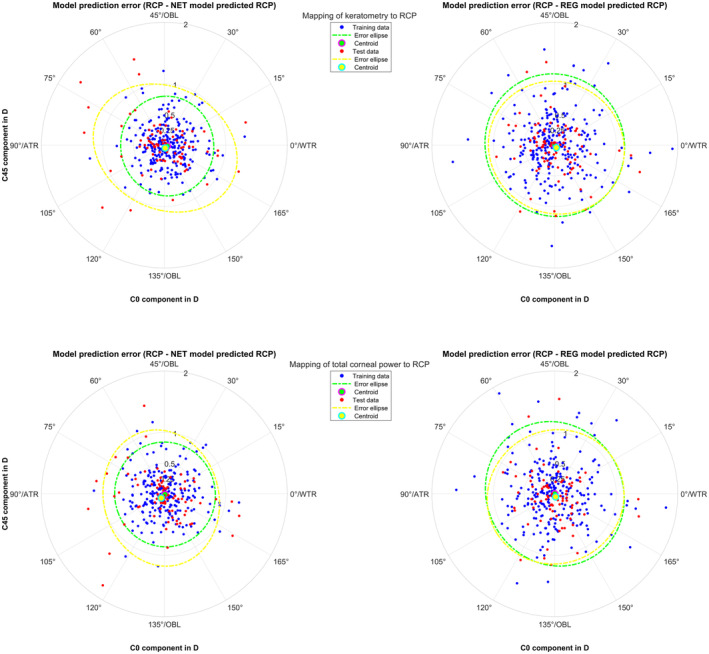
Double angle plot showing the astigmatism power vector components (C0 and C45: Projection to the 0/90° and to the 45°/135° meridian) for the differences between the reconstructed corneal power RCP and the model predictions (prediction error). In the upper/lower left graph the prediction error is shown for the neural network based predictions from preoperative keratometry/total corneal power. In the upper/lower right graph the prediction error is shown for the multilinear regression from preoperative keratometry/total corneal power. The error ellipses (green dashed lines for the training data and yellow dashed lines for the test data) together with the green and yellow filled circle marker refer to the 95% error ellipses and the centroids of the bivariate distributions. WTR/ATR/OBL respectively refers to the orientation of astigmatism with‐the‐rule/against‐the‐rule/oblique.

The upper part of Table 4 shows the definition of the multivariate linear regression‐based prediction models which map the 3 power vector components from preoperative keratometry and total corneal power to RCP. The 3 × 3 matrices which describe the weights for the input parameters both show a structure of diagonal dominant matrices with elements close to 1 at the diagonal and 0 otherwise. This indicates that the prediction models involve mostly a 1:1 mapping from keratometry or total corneal power to RCP (diagonal elements) without significant crosstalk between the power vector components (off‐diagonal elements). The LogL values refer to the log likelihood objective function at the last iteration and confirm a good performance of the multilinear regression models. All prediction models were trained with the training data and evaluated separately with the training and the test data to evaluate potential overfitting. These results are summarised in the lower part of Table 4. As expected, the regression‐based models yield centroid coordinates at the origin for the training data. The prediction performance in terms of MDV, MSE and area of the error ellipses for both NET models systematically outperform the respective REG models for the training data. However, for the test data MDV, MSE and area of the error ellipses are quite similar with the NET and REG prediction models. This means that especially in both NET prediction models with the lower performance in the test data compared to the training data we notice some overfitting. Figure 2 displays the respective astigmatism power vector components C0 and C45 for the model prediction error based on preoperative and postoperative keratometry (upper graphs) and preoperative and postoperative total corneal power (lower graphs) for the training and the test data. Again, we see from the data scatter and the error ellipse areas in the graphs that the 2 NET prediction models (left graphs) outperform the respective REG prediction models (right graphs), especially for the training data. However, with crossvalidation we see that both NET prediction models overfit and that the performance in the test data is quite similar with the NET and REG prediction models.

**TABLE 4:** Upper part: Definitions of the multilinear regression based prediction models (REG) for mapping the vector components of preoperative keratometry and total corneal power to the respective power vector components of reconstructed corneal power RCP. Keratometry was derived from the IOLMaster 700. logL refers to the log likelihood objective function at the last iteration. Lower part: Listing of the centroid coordinates in dioptres, area of the error ellipse indicating the 95% confidence region in square dioptres as a measure of data scatter, mean difference vector MDV in dioptres, and mean squared prediction error MSE in square dioptres for the 2 REG and NET prediction models. As expected, the regression based models yield centroid coordinates at the origin for the training data.

*N* = 442 Data in dioptres		Equation of the multivariate linear regression models REG									logL
Multilinear regression model REG	RCP prediction from keratometry	$\text{Predicted}\;\left[\begin{array}{c} \mathrm{EQ}\\ C0\\ C45 \end{array}\right]=\left[\begin{array}{ccc} 0.9458 & 0.0332 & -0.0104\\ 0.0009 & 0.9135 & -0.0711\\ 0.0129 & 0.0315 & 0.8557 \end{array}\right]∙\left[\begin{array}{c} \mathrm{EQ}\\ C0\\ C45 \end{array}\right]+\left[\begin{array}{c} 2.5173\\ -0.1031\\ -0.5451 \end{array}\right]$									−552
	RCP prediction from total corneal power	$\text{Predicted}\;\left[\begin{array}{c} \mathrm{EQ}\\ C0\\ C45 \end{array}\right]=\left[\begin{array}{ccc} 0.9523 & 0.0360 & 0.0011\\ -0.0196 & 0.8931 & -0.0342\\ 0.0125 & -0.0424 & 0.8148 \end{array}\right]∙\left[\begin{array}{c} \mathrm{EQ}\\ C0\\ C45 \end{array}\right]+\left[\begin{array}{c} 2.1739\\ 0.9693\\ -0.4971 \end{array}\right]$									−529
	Model prediction errors (predicted RCP – calculated RCP)	Training data (*N* = 328)					Test data (*N* = 140)				
		Centroid X	Centroid Y	Error ellipse area in D^2^	MDV	MSE	Centroid X	Centroid Y	Error ellipse area in D^2^	MDV	MSE
	RCP prediction from keratometry	0.0000	0.0000	4.1416	0.4014	0.2176	0.0254	−0.0400	3.7523	0.3834	0.2158
	RCP prediction from total corneal power	0.0000	0.0000	4.1633	0.4017	0.2163	0.0132	−0.0491	3.8198	0.3767	0.2152
Feedforward neural network NET	RCP prediction from keratometry	0.0465	−0.0141	1.9314	0.2886	0.1100	0.0131	−0.04699	3.7748	0.3679	0.2010
	RCP prediction from total corneal power	0.0103	−0.00997	2.1930	0.3029	0.1198	−0.0506	−0.0685	3.2917	0.3443	0.1757

**FIGURE 3:**
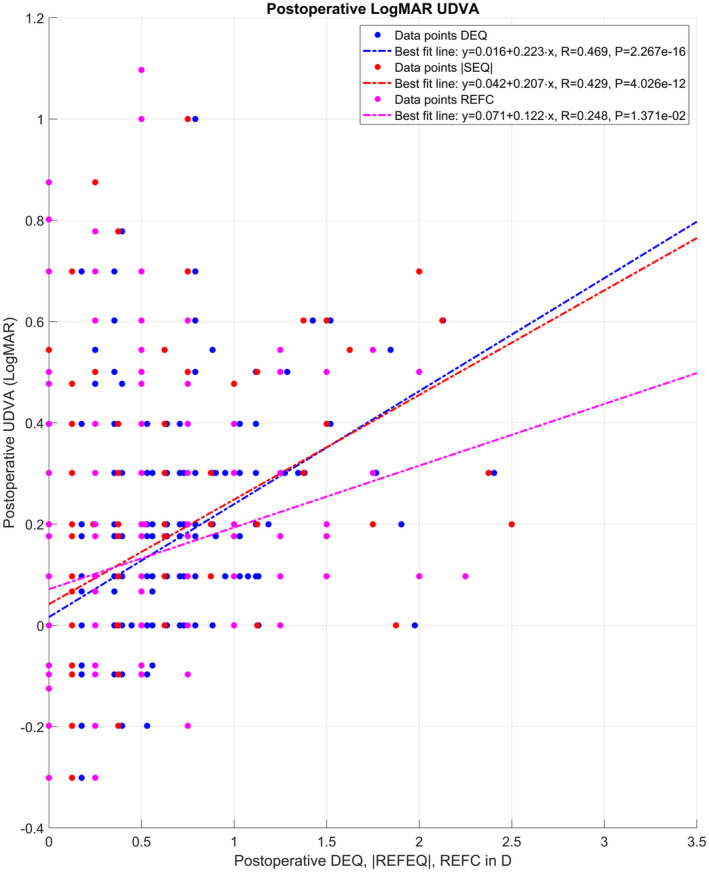
Scatterplot showing the correlation of the postoperative uncorrected distance visual acuity UDVA to the defocus equivalent DEQ, the absolute value of the spherical equivalent of refraction |REFEQ|, and the refractive cylinder REFC. The graph indicates that UDVA decays with increasing DEQ, |REFEQ| and with REFC. From the best fit lines shown in the figure legend it can be seen that for each dioptre of DEQ/|REFEQ|/REFC the expected decay of LogMAR UDVA is 2.2/2.1/1.2 lines. The respective correlation coefficients R and significance levels P are as listed in the legend of the graph.

Figure 3 refers to eyes with implantation of the monofocal toric lens (AT TORBI, *N* = 282) and shows a scatterplot for the uncorrected distance visual acuity UDVA as a function of the postoperative defocus equivalent DEQ, the spherical equivalent error |REFEQ|, and the refractive cylinder REFC. The graph indicates that after toric lens implantation UDVA systematically decays with the residual refractive error in terms of DEQ, |REFEQ| and REFC. From the best fit lines shown in the figure legend, we see that for each dpt of DEQ/|REFEQ|/REFC the expected decay of LogMAR UDVA is 2.2/2.1/1.2 decimal lines.

**FIGURE 4:**
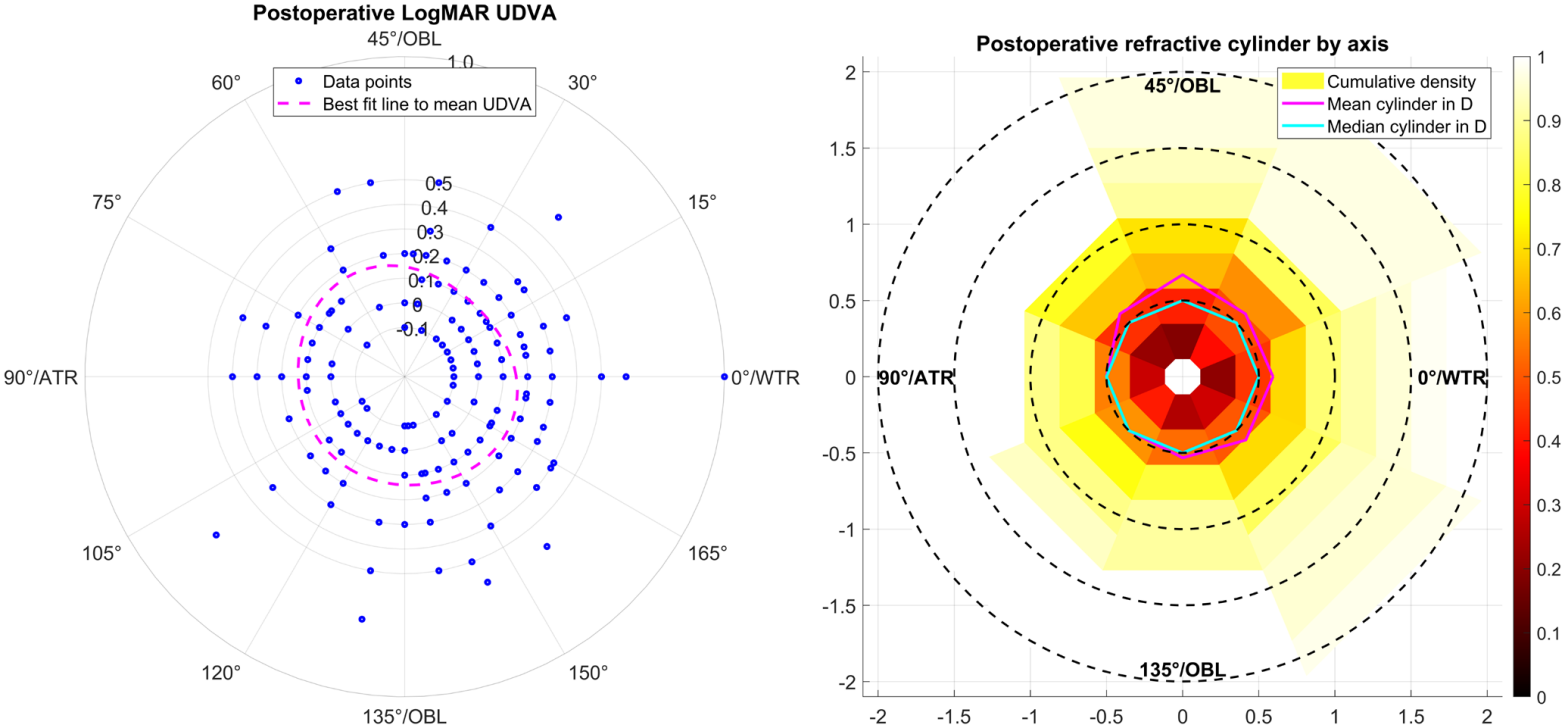
Left graph: Scatterplot showing the correlation of the uncorrected distance visual acuity UDVA as a function of the axis of residual refractive cylinder REFA after cataract surgery with implantation of a monofocal toric lens. The magenta dashed line refers to the mean UDVA over REFA and shows only slight variations in the axis range from 0 to 180°. Right graph: Heat map showing the cumulative density function of the refractive cylinder for different 8 equally spaced bins of REFA range from 0 to 180°. The graph indicates that in the axis range from 60 to 150° for right eyes (30–120° for left eyes) we measured only refractive cylinder values up to 1.25 dioptres, whereas for the remaining axis range the residual refractive cylinder could take larger values. The magenta and cyan lines refer to the mean and median refractive cylinder for each bin respectively.

The left graph of Figure 4 refers to the subpopulation of eyes (with implantation of the monofocal toric lens (AT TORBI) and a refractive cylinder REFC ≥0.25 D; *N* = 186) and shows the UDVA (radial direction) as a function of the axis of the postoperative refractive cylinder REFA (azimuthal direction). The best fit line (magenta dashed line) gives us the mean UDVA as a function of REFA indicating that the UDVA shows only slight variations over REFA with a mean value between 0.1 to 0.2 LogMAR. The right graph refers to eyes with a residual refractive cylinder REFC ≥0.25 D (*N* = 256) and shows the heatmap of the normalised cumulative density for the postoperative refractive cylinder by axis. The axis range from 0 to 180 degree was divided into 8 equidistant slots. From the heatmap, we see that, especially in the slot around 45° ± 15° (for left eyes: 135° ± 15°), some clinically relevant residual refractive cylinder is measured, whereas in the range from 75° to 150° (for left eyes: 30° to 105°) the residual refractive cylinder is within limits of 1.25 dioptres. The median refractive cylinder as indicated by the cyan line is 0.5 dioptres for all slots.

## 4 DISCUSSION

There is considerable controversial discussion in the literature as to which measure or combination of measures should be used for toric lens power calculation. The options include keratometric values used alone, keratometric values with some statistical correction for the posterior corneal astigmatism, or measurement data from both corneal surfaces from a tomographer. For tomographic data, we could select either a thick lens model for the cornea with separate data on the anterior and posterior corneal surface or a thin lens model with compound data which represent the thick lens cornea as an equivalent thin replacement lens (Lu et al., 2019; Park et al., 2017; Patel & Tutchenko, 2021; Preussner et al., 2015). Since Javal we are aware that keratometry which measures only the anterior corneal curvature does not fully represent corneal power, especially corneal astigmatism (Elliott et al., 1994). Where a tomographer or biometer with the options of posterior corneal measurement is not available, we are restricted to keratometric data (Tutchenko et al., 2020). To account for the posterior corneal astigmatism 3 different concepts for a nomogram correction are established: first we could map preoperative keratometry to preoperative total corneal power in a representative dataset; second we could map preoperative keratometry to postoperative total corneal power; and third we could map preoperative keratometry to RCP back‐calculated from postoperative refraction and toric lens power and postoperatively measured axis.

With the first option, we are restricted to a preoperative ‘snapshot of corneal power’, and potential changes in corneal geometry could be considered separately with a statistical correction of SIA (Langenbucher, Hoffmann, et al., 2024). The second option also considers the (deterministic portion of) changes in the cornea from the preoperative to the postoperative state. The third option additionally considers the deterministic portion of any mismatch between corneal astigmatism and refractive cylinder, for example, resulting from a lack of coaxial alignment of the cornea and the IOL to the visual axis. However, all these options have advantages and drawbacks: the first option requires a separate evaluation (and potentially a correction) of SIA, which is not required for the second or third option. Conversely, options 2 and 3 include changes in the corneal geometry from preoperative to postoperative and therefore they depend on the surgical technique. In addition, the third option depends mainly on the quality of the postoperative refraction. This means that postoperative refractometry requires special care and autorefractometry cannot be recommended. It also reflects the subjective cortical perception of astigmatism, something none of the alternative methods do (Goggin et al., 2022).

In the present study, we implemented a concept based on the third option which maps the 3 power vector components of preoperative keratometry and total corneal power to the reconstructed corneal power RCP. Since our dataset includes reliable manual refraction data and measurement of the toric IOL orientation based on slitlamp examination, this allowed us to derive models to predict the respective power vector components of RCP. For the mapping, we implemented 2 different concepts: a simple feedforward shallow neural network with 2 hidden layers and a multilinear regression‐based prediction model. The feedforward neural network structure was used because it can easily map multiple input data to multiple output data (Sheen Ophir et al., 2020), which is not the case for many other neural network structures. The multilinear regression‐based prediction has the advantage of easy implementation e.g. in consumer software such as Microsoft Excel (Meng et al., 1993; Sexton & Swensen, 2000). The entire dataset was randomly split into training and test data, and all prediction models were defined and trained based on the training data. The evaluation of the prediction models was performed using both the training and the test data. For the training (and defining the weights and biases) the MSE quality metric was used. This metric is a common choice for the performance of regression‐based prediction models. The multilinear regression models were optimised for the Log likelihood performance metric as commonly used for robust regression estimators such as the ECM method (Sexton & Swensen, 2000). Since our output data consists of 3 vector components, we used the generalised version of the mean squared error MSE based on the Euclidean norm (Langenbucher, Szentmáry, Cayless, Weisensee, et al., 2022).

Our results indicate that the mean corneal equivalent power EQ of preoperative and postoperative keratometry and total corneal power appear quite similar as shown in Table 2. The preoperative and postoperative C0 component of total corneal power appears systematically more negative compared to the respective C0 components of keratometry, which reflects the ATR contribution of the posterior cornel surface. Based on reported the findings in the literature that left and right eyes show some symmetry in astigmatism with respect to the vertical axis, we consequently flipped the sign of the C45 components of left eyes to treat all eyes in the dataset as right eyes. We observed that the C45 components of preoperative and postoperative keratometry and total corneal power are on average all below zero.

From the differences in the astigmatic power vector components between RCP and the measured preoperative and postoperative keratometry and total corneal power as shown in Figure 1, we find that the RCP matches well with the postoperative total corneal power (area of the error ellipse 3.52 D^2^) and somewhat less well with the preoperative keratometry (area of the error ellipse 4.89 D^2^), postoperative total corneal power (area of the error ellipse 5.02 D^2^) and postoperative keratometry (area of the error ellipse 5.05 D^2^). This result is not immediately foreseeable as we used the predicted axial lens position according to the Haigis formula which might have some inaccuracies. Also, the labelled equivalent and toric power of the lens may show some variation according to ISO 11979. Furthermore, the toric lens orientation derived from postoperative slitlamp images could be subject to some measurement noise and the postoperative refractometry could again show some measurement noise. From Table 3 which lists the difference data for all 3 power vector components we understand that on average, the C0 component of RCP slightly overestimates the respective value for preoperative and postoperative total corneal power (0.06 and 0.04 D), but it systematically underestimates preoperative and postoperative keratometry (−0.14 and −0.12 D).

However, the most important finding in this study is that based on our data, both variations of the Homburg‐Adelaide toric IOL nomogram, the feedforward neural network and the multilinear regression models all provide a good prediction for the RCP based on preoperative keratometry and total corneal power as shown in Table 4. Using the training data, we observed a mean difference vector of 0.29 D and 0.30 D when predicting RCP from preoperative keratometry and total corneal power with the neural network setup. The respective mean difference vector was 0.40/0.40 D with the multilinear regression setup. During crossvalidation with the test data, we observed a mean difference vector of 0.37/0.34 D with the neural network, and 0.38/0.38 D with the multilinear regression. Especially when considering the mean squared prediction error MSE as a quality metric to assess the performance of our prediction models, we see that the neural network outperforms the multilinear regression only for the training data, but not systematically for the test data. For mapping of keratometry/total corneal power to RCP the NET performed with a MSE = 0.11/0.12 D^2^ when used on the training data compared to 0.20/0.18 D^2^ with the test data. The REG showed similar performance on both the training data (MSE = 0.22/0.22 D^2^) and the test data (MSE = 0.22/0.22 D^2^). This implies that the shallow feedforward neural network used in this study has an excellent performance primarily with the training data, but because of overfitting the real performance on an independent dataset might be comparable to that of the multilinear regression. For a use in a clinical environment, the multilinear regression models as shown in the upper part of Table 4 are in any case much easier to implement. In situations where only predictions of the astigmatism power vector components are required, the models could easily be simplified.

After tIOL implantation the UDVA seems to be correlated with the residual spherocylindrical refraction error. The scatterplot in Figure 3 shows that after cataract surgery with implantation of a toric lens, the uncorrected visual acuity decays by around 2.2 lines for each dioptre of DEQ. For the mean refractive equivalent error |REFSEQ| and the mean refractive cylinder REFC the respective decays are 2.1 and 1.2 lines per dioptre. This decay of uncorrected visual acuity over |REFEQ| and REFC is in accordance with the literature data (Blendowske, 2015; Raasch, 1995) for lower values of DEQ < 2 D, but it does not fully match the classical rule of thumb in ophthalmology that in absence of accommodation a spherical or cylindrical refraction error of 1 D degrades uncorrected vision by 3 or 1.5 decimal lines. These data indicate that in cases with a clinically relevant preexisting corneal astigmatism we should always aim for correction with a tIOL in case of cataract surgery. From the left graph in Figure 4, we learn that the distribution of UDVA shows no systematic variation for the axis of the refractive cylinder in a subpopulation of eyes treated with a monofocal toric lens showing some residual refractive cylinder (≥0.25 D). The mean LogMAR UDVA varies between 0.1 and 0.2 for the entire range of REFA, which means that there is no individual axis corresponding to a greater degradation in uncorrected vision. On the right graph the cumulative density heatmap of the residual refractive cylinder (monofocal or multifocal lenses, REFC ≥0.25 D) gives some detailed insight into the distribution of the amount and axis of the refractive cylinder. We can see directly from the graph that for axis orientations from 150° through 180°/0° to 60° for right eyes (or from 30° to 120° for left eyes) we measured values for the residual refractive cylinder up to 2 D, whereas in the residual axis range the maximum of the residual refractive cylinder is ≤1.25 D. This phenomenon should be investigated more in detail with a larger study population.

However, this study shows some limitations: first, the study was restricted to a nomogram correction mapping the keratometry and total corneal power to the reconstructed corneal power RCP. The correction is based on IOLMaster 700 measurements and cannot be generalised to keratometric or total corneal power data from other instruments (Park et al., 2017; Patel & Tutchenko, 2021; Preussner et al., 2015; Savini et al., 2017; Tutchenko et al., 2020; Wendelstein et al., 2023); Second, we included the monofocal and the trifocal model of the toric Zeiss lens in our study, and these models may perform differently in the eye. The analysis of the decay of UDVA as a function of the residual refraction error was restricted to the monofocal toric lens model. Third, the quality of postoperative refractometry, the power data of the tIOL, the prediction of the axial tIOL position and the evaluation of the tIOL orientation are all crucial when back‐calculating the RCP. All these data could be subject to some measurement noise which might affect the RCP and therefore our prediction models. And finally, the choice of mapping preoperative keratometry and total corneal power to RCP means that the potential deterministic portion of the change in corneal geometry is already included in the prediction model. This means that the prediction models would need to be adapted for use with different surgical techniques or corneal incisions which induce corneal astigmatism.

In conclusion, our results indicate that the 3 power vector components of preoperative IOLMaster 700 keratometry and total power could be properly mapped to the reconstructed corneal power RCP after cataract surgery with toric lens implantation. The RCP matches better to postoperative total corneal power than to preoperative or postoperative keratometry or preoperative total corneal power. The neural network and the multilinear regression‐based prediction models which map preoperative keratometry and total corneal power to RCP generally show a good performance. The multilinear regression models in particular represent a solution for predicting RCP as a measure for the corneal power after cataract surgery that could easily be implemented using consumer software and used in toric lens power calculation in a clinical setting.

## CONFLICT OF INTEREST STATEMENT

The authors report no conflicts of interest and have no proprietary interest in any of the materials mentioned in this article. The authors received no specific funding for this work. Prof. Langenbucher reports speaker fees from Hoya Surgical, Johnson & Johnson Vision and Carl Zeiss Meditec outside the submitted work. Prof. Szentmáry and Dr. Cayless report no conflicts of interest. Dr. Wendelstein reports research grants from Carl Zeiss Meditec AG, speaker fees from Carl Zeiss Meditec AG, Alcon, Rayner, Bausch and Lomb, and Johnson & Johnson Vision outside of the submitted work. Dr. Hoffmann reports speaker fees from Hoya Surgical and Johnson & Johnson outside the submitted work. A/Prof. Goggin reports no conflicts of interest.
